# Thyroid nodule ultrasound: technical advances and future horizons

**DOI:** 10.1007/s13244-015-0398-9

**Published:** 2015-03-05

**Authors:** Andrew S. McQueen, Kunwar S. S. Bhatia

**Affiliations:** 1 Freeman Hospital, Newcastle upon Tyne, UK; 2 Department of Imaging & Interventional Radiology, The Chinese University of Hong Kong, Prince of Wales Hospital, Shatin, N.T., Hong Kong

**Keywords:** Thyroid nodules, Thyroid cancer, Ultrasound, Elastography, Malignancy

## Abstract

**Abstract:**

Thyroid nodules are extremely common and the vast majority are non-malignant; therefore the accurate discrimination of a benign lesion from malignancy is challenging. Ultrasound (US) characterisation has become the key component of many thyroid nodule guidelines and is primarily based on the detection of key features by high-resolution US. The thyroid imager should be familiar with the strengths and limitations of this modality and understand the technical factors that create and alter the imaging characteristics. Specific advances in high-resolution US are discussed with reference to individual features of thyroid cancer and benign disease. Potential roles for three-dimensional thyroid ultrasound and computer-aided diagnosis are also considered. The second section provides an overview of current evidence regarding thyroid ultrasound elastography (USE). USE is a novel imaging technique that quantifies tissue elasticity (stiffness) non-invasively and has potential utility because cancers cause tissue stiffening. In recent years, there has been much research into the value of thyroid USE for distinguishing benign and malignant nodules. Preliminary findings from multiple pilot studies and meta-analyses are promising and suggest that USE can augment the anatomical detail provided by high-resolution US. However, a definite role remains controversial and is discussed.

***Teaching points*:**

*• High-resolution US characterises thyroid nodules by demonstration of specific anatomical features*

*• Technical advances heavily influence the key US features of thyroid nodules*

*• Most papillary carcinomas appear stiffer than benign thyroid nodules on US elastography (USE)*

*• Thyroid USE is controversial because of variation in the reported accuracies for malignancy*

*• Combined grey-scale US/USE may lower the FNAC rate in benign nodules*

## Introduction

Ultrasound (US) examination of the neck is a commonly performed investigation and thyroid nodules are a highly prevalent finding. As the vast majority of nodules are non-malignant, the accurate discrimination of a benign from cancerous lesion is an important and challenging feature of thyroid US. Whilst single US features remain of limited accuracy, constellations of findings enable thyroid nodules to be accurately stratified on a ‘risk-of-malignancy’ basis and form the basis of several guidelines [1, 2]. Imaging features that confer a high positive predictive value and specificity for malignancy are valuable as they enable suspicious lesions to be identified and targeted for pathological analysis (typically fine-needle aspiration cytology, FNAC). The ubiquitous nature of thyroid nodules and the limitations of cytology mean that the confident identification of *benign* nodules is also important. US characteristics that carry a high negative predictive value for malignancy enable an evidence-based decision not to investigate further, allowing unnecessary FNAC and/or surgery to be avoided in a very large population. Many of these key thyroid nodule features are heavily dependent on recent advances in high-resolution ultrasound technology and are therefore altered and influenced by US machine settings. In an era of wider US use by different groups (e.g., endocrinologists, surgeons), the high-resolution features of thyroid disease—and the technical factors, which create and alter these valuable signs—must be familiar to the operator.

US elastography (USE) is a recent technological advance that measures tissue elasticity or stiffness properties objectively and has been available in recent years on many state-of-the-art clinical US machines. USE is classified as diagnostic ultrasound and is safe, non-invasive and requires no costly consumables. Importantly, USE can be performed in real-time alongside conventional sonography, providing objective stiffness data that can be used to influence clinical decisions during US examinations. The clinical potential of USE relies on the same principles as clinical palpation, namely that processes such as malignancy and fibrosis alter elasticity. Numerous clinical applications of USE are under investigation at different body sites. To date, over 100 preliminary studies on USE for thyroid nodules for malignancy have been published, with promising but controversial results, as will be discussed.

This article will focus on two aspects of thyroid ultrasound: first, technical advances in high-resolution US are discussed with relevance to specific features of thyroid cancer, benign disease and emerging developments. The second part of the article provides a concise overview of the current role of elastography and how elasticity imaging may supplement high-resolution ultrasound in the pursuit of accurate thyroid nodule assessment.

## Advances in ultrasound image resolution

**Fig. 1:**
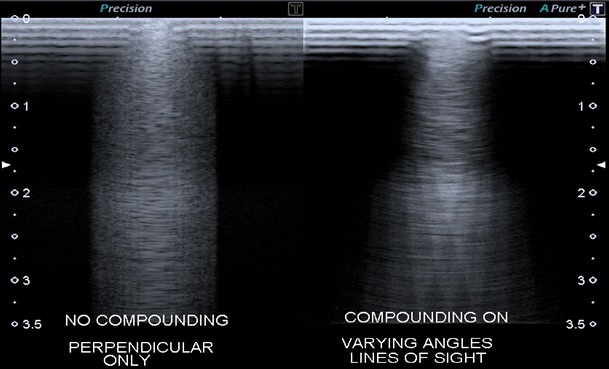
High-resolution images through a phantom using perpendicular (**a**) and spatial compounding (**b**) beams. Courtesy of Toshiba

Thyroid nodule detection, margin delineation and size measurement are heavily influenced by spatial resolution. Modern high-frequency US probes utilise piezoelectric material that resonate at higher frequencies and across wider bandwidths (i.e., PureWave^TM^, Philips) than older crystals. Improving the source piezoelectric material enables the effective bandwidth of the probe to be used more efficiently and uniformly than was previously possible [3]. The resulting improvements in spatial resolution are particularly beneficial in superficial structures such as the thyroid. In addition, probes now contain higher numbers of elements (e.g., high-density 18 L6, Siemens ACUSON) facilitating more versatile beam forming and providing the basis for increasingly advanced compound imaging. Spatial compounding refers to composite images created by insonations from transmitted pulses at multiple different angles to the probe surface (Fig. 1) whilst frequency compounding generates images based on a range of frequencies across the probe bandwidth [4]. The two techniques are often used simultaneously, producing higher resolution images with smooth tissue planes and increased signal-to-noise ratios. Traditionally, these methods of improving spatial resolution have come at a cost: a reduced frame rate (temporal resolution) due to the conventional ultrasound transmit-receive cycle. The newest technical developments aim to minimise this limitation; an example is the use of precision beam-forming, multi-line transmission and parallel processing (nSIGHT^TM^, Philips) to combine high spatial and temporal resolution. As a final consideration, the availability of high-definition, multi-position monitors enables the information acquired and processed by the transducer to be clearly displayed for interpretation.

Contrast resolution settings are the primary factors that influence the assessment of nodule consistency, reflectivity and the detection of echogenic foci due to calcium or colloid. Whilst broader bandwidths allow improved beam penetration in the neck, the majority of advances in this aspect of thyroid ultrasound relate to how the echoes are received and processed rather than the pulse transmission. Signal processing (also known as speckle reduction) techniques are designed to interrogate lines of echoes obtained on each frame and improve the signal-to-noise ratio by pattern recognition [5]. For example, an anechoic area containing background noise will be recognised as a cystic structure and the abrupt change in echoes around the margins of this target then recognised as the cyst/vessel wall (e.g., Precision Imaging^TM^, Toshiba). This process of removing unwanted noise leads to images that look sharper because of the improved signal-to-noise ratio and emphasise cystic vs. solid consistency. The dynamic range and grey-scale map are inherently linked but different aspects of high-resolution ultrasound. The dynamic range controls the range of grey (or the preferred colour tint) that is available to display echo intensity, whilst the grey-scale map allocates which parts of this spectrum are used to represent the echo signals received [6]. Tissue harmonic imaging (THI)—an established technique for improving image contrast at other body sites—has recently become more relevant to high-frequency ultrasound. As the basis of this technique is to receive a signal from the second harmonic frequency of the probe’s transmit pulse (i.e., twice the probe transmit frequency), its use in superficial structures has been limited [7]. However, the advent of wider bandwidths and more sophisticated receive technology means that THI is increasingly available to use with high-frequency probes (e.g., Hitachi High definition dynamic THI) resulting in further removal of clutter/noise and improved tissue contrast. Basic ultrasound settings (focal zones, time gain control, etc.) also affect image quality but are not covered in this article.

**Fig. 2:**
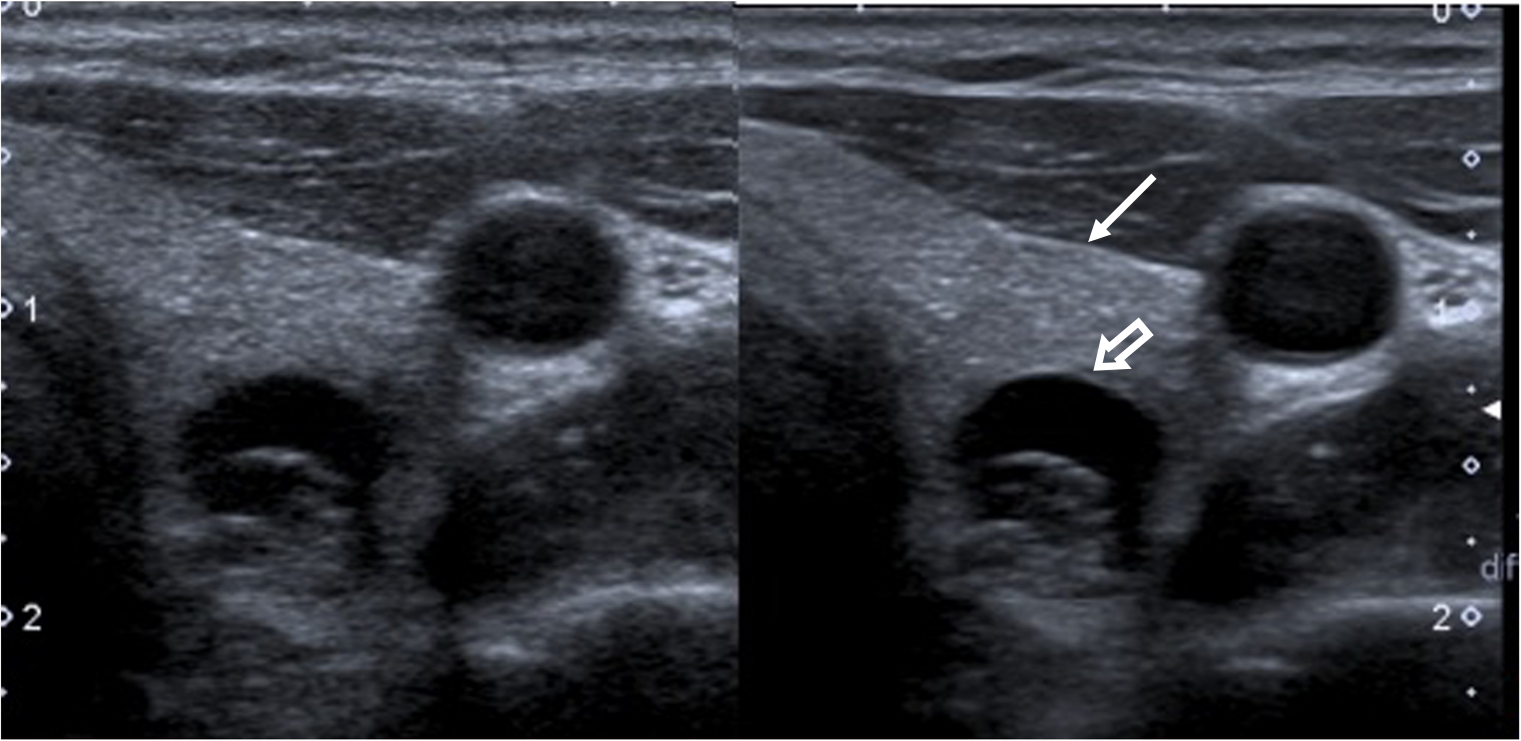
Transverse images of a predominantly cystic left lobe nodule obtained without (*left)* and with *(right*) spatial and frequency compounding software. Note the improved delineation of the thyroid (*arrow*) and cystic nodule (*block arrow*) margins

**Fig. 3:**
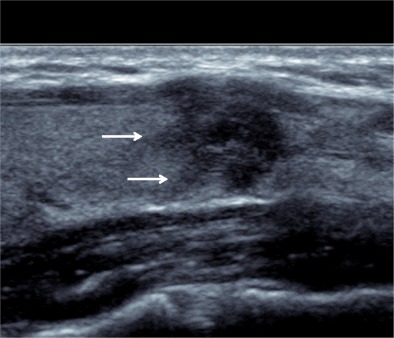
Longitudinal image of a hypoechoic nodule with a spiculated margin (*arrows*) and anterior capsular breach on high-resolution ultrasound. The lesion was confirmed as a papillary thyroid carcinoma on surgical resection

The following key thyroid nodule characteristics are heavily influenced by these improvements in image resolution. Whilst multiple factors contribute to the assessment of these signs, the predominant technical consideration is discussed for each in turn:

- Margin delineation:

An abnormal thyroid nodule margin is a recognised feature of thyroid malignancy. Assessment of the nodule edge has previously been binary; the margin is either well or poorly defined. A poorly defined/irregular nodule margin conferred 87 % specificity for malignancy in a study population of 1,108 thyroid nodules but with a very low sensitivity of 39 % [8]. Whilst operator experience is an important factor in edge delineation, the machine settings heavily influence this assessment and create a challenge for standardised reporting between observers using different equipment. The probe frequency, bandwidth and use of compound imaging are the key technical aspects to consider here (Fig. 2), delivering improved signal-to-noise ratios for the operator to delineate the nodule from the surrounding thyroid parenchyma and adjacent structures. More recently, the concept of a ‘spiculated’ margin (Fig. 3) has been introduced to describe the finding of a distinctly infiltrative nodule edge (as separate from the broader ‘poorly defined’ category) with 92 % specificity and 81 % positive predictive value in a comprehensive review of B mode ultrasound findings [9]. The ability to detect margin spiculation is a consequence of higher spatial and temporal resolution and it therefore follows that the reliable detection of this useful characteristic requires access to the advances in the above-described ultrasound technology. Understanding the close relationship of technology to sonographic interpretation is particularly important because—of all the individual thyroid nodule features—margin delineation has the poorest inter-observer agreement [10]. The multiple technical factors are likely to contribute to this variability and are particularly relevant when patients undergo thyroid ultrasound at different centres with varying ultrasound probe and software settings.

**Fig. 4:**
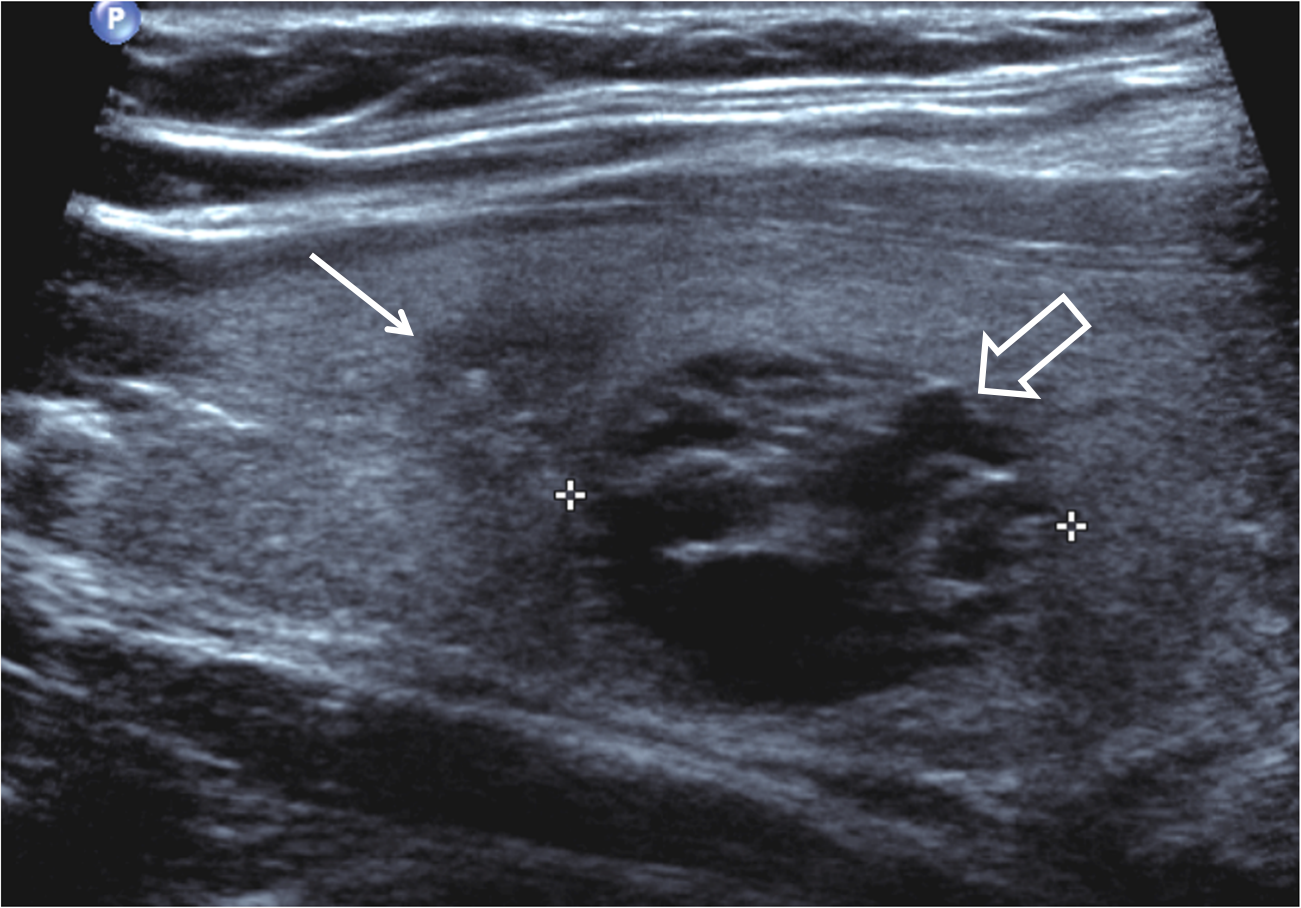
Longitudinal image of a 1.5-cm nodule (*block arrow*) detected incidentally on cervical spine MRI. This nodule is spongiform and appears confidently benign. However, a 0.6-cm ill-defined nodule alongside (*arrow*) is hypoechoic and contains microcalcification—FNA was performed from this nodule and confirmed papillary thyroid carcinoma

- Smaller nodule detection:

The rising incidence of thyroid cancer across many countries is almost entirely attributable to small (<2 cm) papillary carcinoma [11]. Multiple factors have contributed to the phenomenon of the clinically occult papillary thyroid cancer; incidental detection on other imaging modalities, patient awareness and access to health care amongst others. The ability to visualise and identify suspicious features within these small nodules (e.g., margin irregularity, microcalcification) is, however, a consequence of the advances in high-resolution ultrasound. Sub-centimetre nodules can be identified, characterised and targeted for FNA if appropriate (Fig. 4). This has created difficulty for guidelines that have been primarily based on nodule size (i.e., the Society of Radiologists in Ultrasound [12]), particularly given the increasing understanding that size alone is not an accurate predictor of malignancy [13]. Moreover, improved resolution enables better needle tip visualisation during US-guided FNA/biopsy and obtaining an adequate cytological specimen from very small (i.e., 5 mm) nodules is feasible, although lower adequacy rates are reported from FNA of these smallest lesions [13]. The clinical benefit of confirming malignancy in this setting is controversial—and beyond the scope of this article—as the prognosis for small papillary thyroid cancers is generally excellent, prompting the proposal of a separate nomenclature, ‘papillary lesion of indolent course’ [11]. The reality for the thyroid US operator at present is that he or she will regularly be confronted with small but suspicious incidental nodules that would previously have remained undetected.

- Nodule consistency

The majority of malignant nodules are solid, but the majority of solid nodules are benign. Whilst solid nodule consistency is of very limited value in identifying thyroid cancer, the finding of microcystic aggregates creating a ‘spongiform’ appearance is an extremely strong predictor of benignity (99.7 % negative predictive value) [9]. As purely cystic nodules are also confidently benign, the ability to accurately detect cystic spaces within a nodule is an important facet of the ultrasound assessment. Spongiform areas can be detected with the aid of compound imaging whilst speckle reduction software removes the noise/clutter from the small cystic spaces. Without the use of these techniques, speckle artefact and blurred septations can mimic solid components and lead to unnecessary investigation of benign nodules.

- Nodule reflectivity

**Fig. 5:**
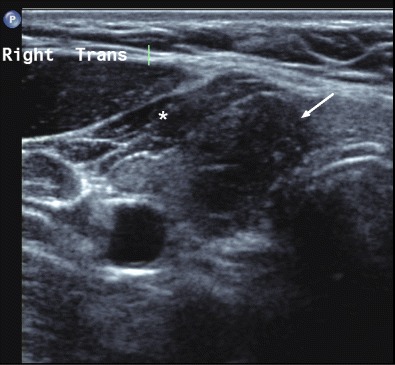
Transverse image of the right thyroid lobe showing a nodule (*arrow)* of slightly lower reflectivity than the adjacent strap muscle (*), i.e., ‘markedly hypoechoic’. A pT1b infiltrative, classical papillary thyroid carcinoma was found on surgical resection

Most thyroid cancers are hypoechoic compared to the background parenchyma. Unfortunately, this finding is non-specific as benign nodules can also be hyporeflective and the comparison with ‘normal’ thyroid may be hampered by background nodularity or thyroiditis. By comparison, marked hypoechogenicity—defined as ‘less reflective than adjacent strap muscle’—is a specific malignant finding with an odds ratio of 8.46 in a retrospective study of 849 thyroid nodules [14]. Although this feature is only seen in the minority of cancers, its recognition makes a benign nodule unlikely and will typically initiate further investigation with FNAC (Fig. 5). Dynamic range and grey-scale map settings predominantly control image contrast whilst the use of harmonic imaging will further emphasise differences in reflectivity. A narrow dynamic range will increase the contrast between adjacent structures by utilising fewer shades of grey to display the echo signal intensity. Whilst these settings are typically configured within manufacturer presets, they can be altered easily on most ultrasound platforms and should ideally be customised to the user’s preferences. The clinical value of marked hypoechogenicity as a specific sign of thyroid cancer emphasises the importance of being familiar with one’s own machine settings.

- Echogenic foci—colloid vs. calcium

**Fig. 6:**
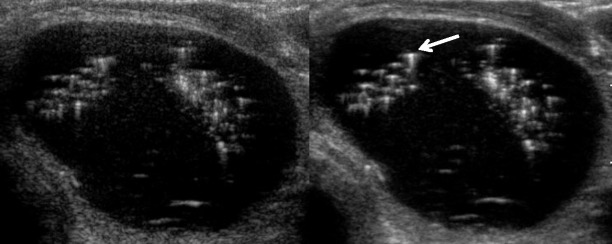
A cystic nodule without (*left*) and with (*right*) signal processing software active. Note the increased conspicuity of the comet tail artefact (*arrow*) on the active image. Images courtesy of Dr S.T. Elliott

In addition to the choice of the dynamic range and grey-scale map, signal processing techniques help to identify small hyperechoic foci. Bright spots that show a ‘ring down’ or ‘comet tail’ artefact are a very helpful finding; they represent foci of inspissated colloid and are a feature of benign nodules. Conversely, the presence of sub-millimetre, highly reflective foci with or without acoustic shadowing represents microcalcification—a specific feature of differentiated thyroid malignancy [15]. The depiction of the comet tail artefact is therefore crucially important to confirm the benign nature of an echogenic focus. As would be expected, signal processing emphasises the bright focus and can help to make the comet tail more conspicuous to the operator (Fig. 6).

**Fig. 7:**
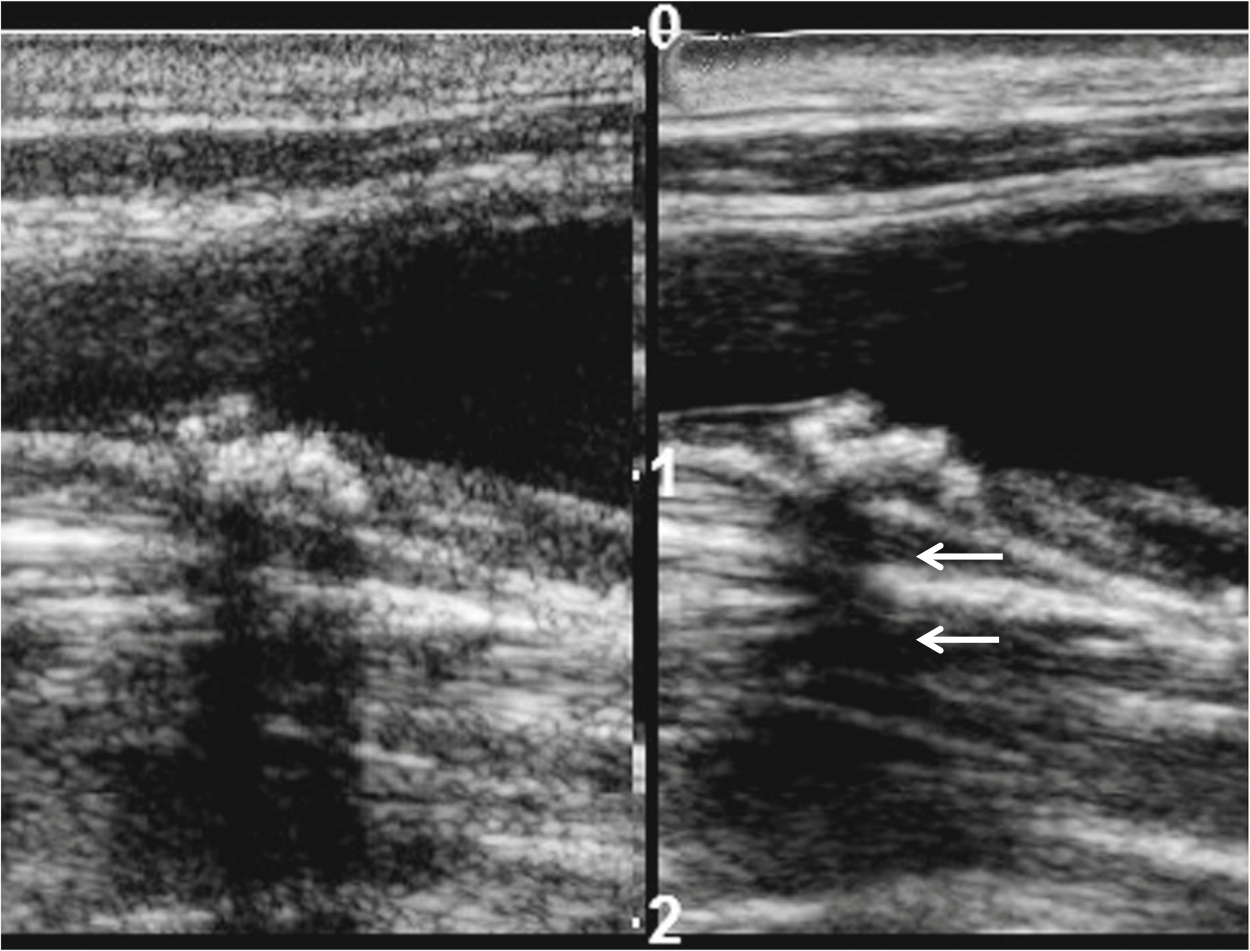
Longitudinal images of carotid bulb atheroma without (*left*) and with (*right)* spatial compounding active. Note the diminished acoustic shadow with spatial compounding (*arrows*) despite the overlying coarse calcification. Images courtesy of Dr S.T. Elliott

Traditionally, the detection of microcalcification (usually due to small psammoma bodies within papillary thyroid cancer) was made easier by the associated acoustic shadow artefact created by reflection of the perpendicular ultrasound beam [16]. In high-resolution ultrasound, the use of spatial compounding means that the signal can be received from tissue deep to calcific foci (Fig. 7) and this artefact is often diminished or lost in the thyroid. This unwanted consequence of modern technology can be overridden by deactivating spatial compounding and allowing acoustic shadows to emerge—if the operator understands the significance of the artefact and its causation.

**Fig. 8:**
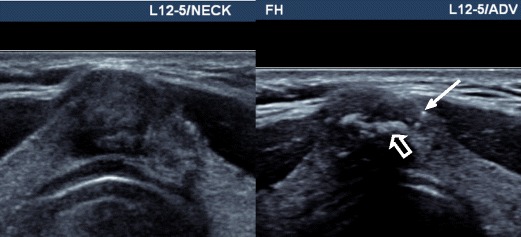
Transverse images of a 1-cm thyroid isthmus nodule using standard thyroid ultrasound (*left*) and modified grey-scale map/dynamic range presets (*right*). Macro- (*block arrow*) and microcalcifications (arrow) are much more conspicuous with the modified preset, originally designed to detect calcification in breast lesions. A follicular variant papillary thyroid carcinoma containing dystrophic and psammomatoid calcification was confirmed on resection

An alternative method of highlighting microcalcification within thyroid nodules is to alter the grey-scale map, dynamic range and speckle reduction combination (Fig. 8). Boosting the brightness of the signal received from calcific spots relative to normal parenchyma increases the detection and has been investigated in breast ultrasound (where the identification of microcalcification also carries significant clinical value) [17]. A novel modified grey-scale map/image tint combination to detect microcalcification (MicroPure Imaging, Toshiba) has been recently reported in thyroid nodules [18].

## Emerging uses of thyroid ultrasound

The technological progress of B mode US enables the thyroid gland to be assessed in more anatomic detail than ever before, characterising lesions by a matrix of structural features. Building on these advances, the value of three-dimensional US in thyroid nodule assessment has recently been considered [19–21]. Initial studies have focussed on two aspects: the ability to more accurately assess the key B mode features (e.g., nodule margin) and the potential to uncover independent predictors of malignancy from 3D data sets. Whilst 3D US is not widely used in the neck at present, recent developments in this area suggest the potential to address persisting challenges of thyroid imaging.

In a prospective study of 91 thyroid nodules, the interobserver agreement for specific characteristics (e.g., shape, margin) was better for 3D than 2D US. Although intra-observer agreement remained suboptimal, there was significantly stronger agreement *between* users for suspicion of malignancy after off-line review of the static 3D images [21]. National guidelines are increasingly based on standardised classification of malignant risk [1, 22] (as opposed to subjective assessment by experienced individuals); therefore reducing inter-observer variation is particularly relevant [1, 21, 22].

In a retrospective study of 71 thyroid nodules, 3D ultrasound data were analysed using multiplanar reformatted (MPR) images and a thin-slice volume-rendered technique [19]. The MPR data found that poorly defined 3D nodule margins were associated with malignancy but also described two novel, independent 3D predictors of malignancy: a lobulated nodule shape in the C-plane (coronal) and altered central 3D vascularity. The smooth surface, thin-slice volume-rendering algorithm created images with higher contrast and reduced noise: a new layer of post processing to compare with existing 2D techniques. The shift in emphasis from real-time image interpretation to volume acquisition with subsequent processing and multiplanar viewing also offers potentially more detailed data analysis. A study of 20 thyroid nodules assessed 3D high-resolution ultrasound data with a computer-aided diagnostics (CAD) software programme [20]. The authors found that CAD-detected specific 3D textural features could be identified and combined as an accurate discriminator of benign and malignant nodules, creating automated malignant risk stratification (‘Thyroid Malignancy Index’). Such an approach would represent a paradigm shift in practice for most thyroid imagers but the potential for accurate objective data to support an operator’s skill and experience is attractive.

Regarding contrast-enhanced ultrasound (CEUS), a small number of studies have documented the feasibility and diagnostic accuracy of assessing thyroid nodule enhancement following intravenous microbubble contrast, using low mechanical index (MI) settings [23, 24]. Recognisable quantitative patterns of perfusion have been identified to construct CEUS malignant risk stratification with 76.9 % sensitivity and 84.8 % specificity in a study of 42 patients [24]. These findings suggest a potential role for CEUS as a diagnostic adjunct to B mode US but the technique is more invasive and time-consuming than standard ultrasound and, at present, the specific clinical utility is unclear.

## US elastography (USE)

- Basic principles

Elasticity refers to the tendency of a tissue to undergo a reversible deformation and is quantified by Young’s modulus (*E*) in kiloPascals (kPa), which equals the ratio of an applied stress to the induced strain (displacement/original length) [25, 26]. USE technologies either estimate elasticity or a surrogate parameter such as strain from US signals acquired during a mild tissue deformation, which can be displayed two-dimensionally as elastograms. A range of proprietary USE technologies is available commercially, which can be divided into strain elastography (strain USE) and shear wave elastography (SWE) based on their underlying physics.

**Fig. 9:**
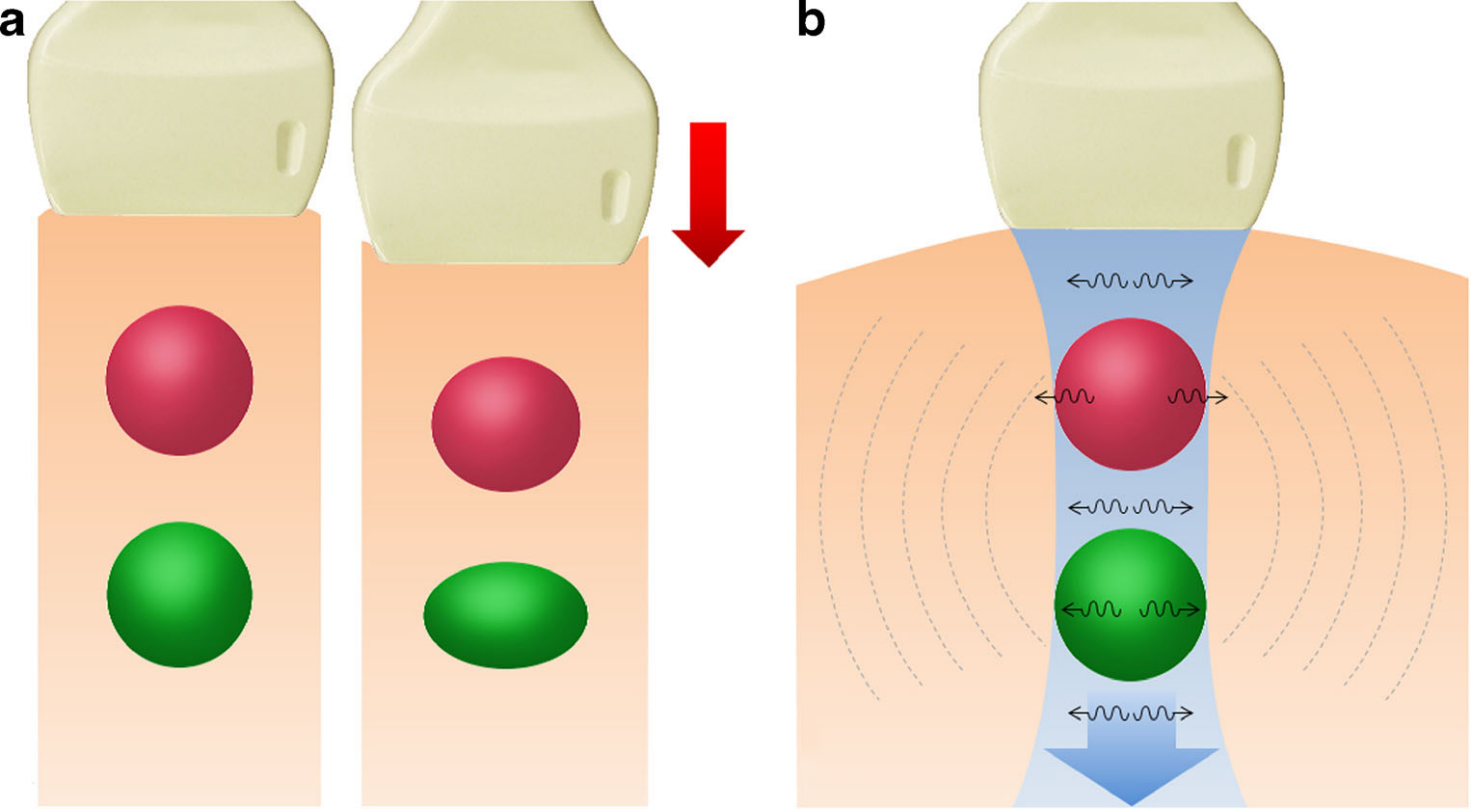
Schematic illustration of (**a**) strain USE and (**b**) SWE for a hard (*red*) and soft (*green*) nodule. In strain USE, tissues are deformed mechanically by the operator via motion of the transducer or by an external source. Relative displacement (*strain*) is greater in soft compared to stiff tissues. In SWE, focused acoustic impulses from the transducer induce laterally propagating shear waves, whose velocities are higher in stiffer tissues. Tissue displacements in both techniques are tracked by ultrasound detection impulses (not shown)

**Fig. 10:**
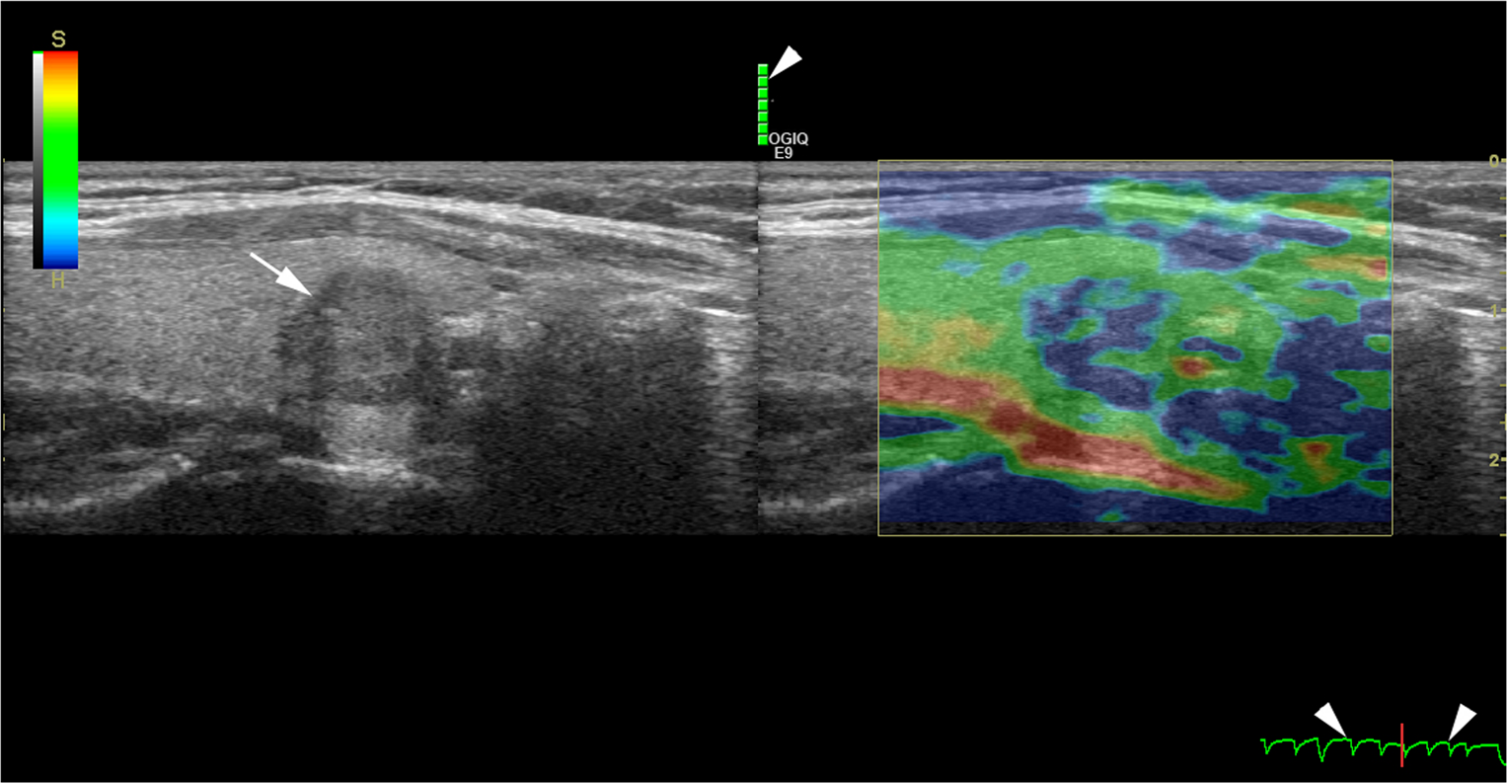
Longitudinal strain USE and B mode US of a papillary carcinoma (*white arrow*). Colour scale in the top left shows stiff, intermediate and soft areas as blue, green and red, respectively. The nodule appears mostly blue suggesting a firm nodule. Compression quality feedback scales are displayed in real time to assist operators in optimising their technique (*arrowheads*)

**Fig. 11:**
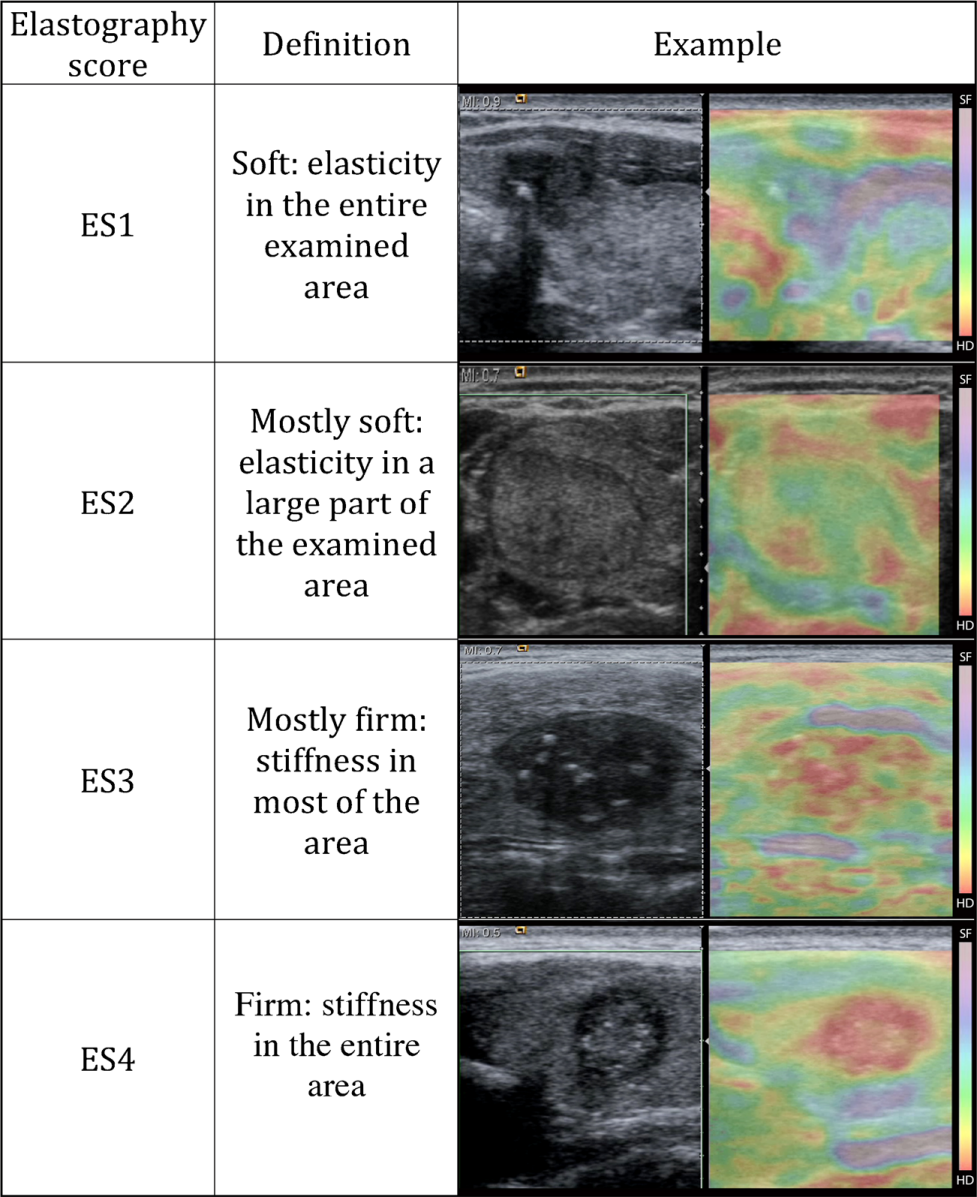
Strain USE scoring system showing a typical 4-point qualitative scoring system. The chromatic scale in this machine, shown on the right side of each elastogram, differs from that used on the machine used to generate Fig. 10, with red and purple colours denoting high and low relative stiffness respectively. The examples shown for ES1 and ES2 were a collapsed haemorrhagic cystic nodule and benign hyperplastic nodule respectively, and ES3 and ES4 were papillary carcinomas. These examples would be classified correctly using most scoring systems published to date, which generally apply a strain USE threshold of ES3 or greater to predict malignancy

In strain USE, also known as quasi-static elastography, compression elastography and real-time elastography (RTE), a mechanical stress is used to deform the tissue of interest and returning US signals are software processed to generate dynamic elastograms of relative tissue strain. In the thyroid, either the operator applies freehand compression to gently compress and decompress a nodule axially under the transducer, or alternatively nodules in the lateral lobe may be compressed solely by transmitted pulsations from the adjacent common carotid artery (Fig. 9). Elastograms are displayed as an opacity layer over and beside corresponding grey-scale US images (split screen mode) using a chromatic scale that varies between manufacturers. Operators performing freehand compression must use a precise manual technique to generate high-quality elastograms, which can be optimised by referring to compression quality scales that are automatically computed and displayed in real time (Fig. 10). Strain elastograms are either interpreted qualitatively, based on visual grading of the proportion and distribution of different colours within the nodule (Fig. 11), or semi-quantitatively using strain ratios (computed from regions of interest (ROIs) placed in the nodule and adjacent thyroid parenchyma) or other indices such as strain heterogeneity. Presently, there is no agreement on an optimal qualitative scoring system although many investigators have used 4- or 5-point scales whereby higher elastography scores (ES) signify higher nodule stiffness [27, 28]. Strain USE is unsuitable for nodules without sufficient reference tissue in the elastogram, which basically excludes single or conglomerate nodules exceeding ~3 cm diameter.

**Fig. 12:**
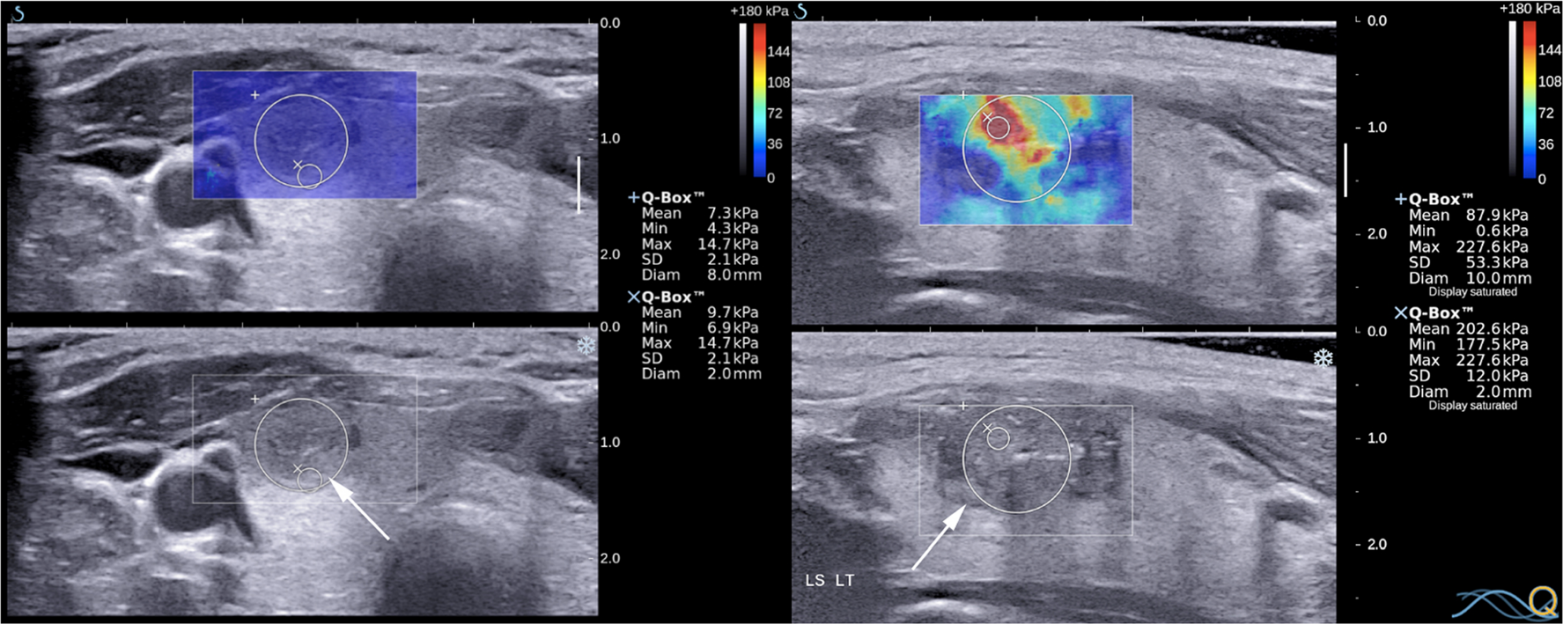
Transverse and longitudinal SWE with B mode US image of a hyperplastic nodule (*left*) and of a papillary carcinoma (*right*) respectively. The SWE elastogram chromatic scale ranges from blue to red, denoting soft to stiff (0 kPa to 180 kPa). Quantitative measurements are acquired for operator-placed ROIs. The benign nodule has a maximum SWE stiffness of 14.7 kPa whereas the cancer has a maximum stiffness of 227.6 kPa

SWE technologies applicable to thyroid imaging use highly focused ultrasound impulses to induce minuscule localised lateral displacements at different locations within the tissue called shear waves, followed by ultrasound detection impulses to track shear wave propagations [25]. SWE is also known as acoustic radiation force impulse imaging (ARFI). Shear waves travel faster in stiffer tissues, whereby shear wave velocity (SWV) is directly proportional to the square root of Young’s modulus. Unlike strain USE, SWE output is quantitative; expressed as SWV (m/s) or estimated tissue stiffness (kPa). Due to differences in proprietary SWE technologies between manufacturers, SWE outputs are varied. These include static colour-coded elastograms termed 2D SWE or a single numerical estimate for an ROI of fixed dimensions (~5 × 6 mm) termed point SWE (pSWE). At least one commercial SWE system produces elastograms in real time, from which SWV or Young’s modulus estimates can be computed for ROIs selected by the operator (Fig. 12).

- Accuracy of USE for malignancy

Presently about 80 studies of strain USE and 20 studies of SWE have been published for thyroid malignancy since 2005 [29–35]. Most are single-institutional series with heterogeneous designs, comprising around 100 nodules selected for FNAC or surgery because of suspicious or equivocal sonographic features, suspicious or malignant cytology or nodules within a compressive goitre. Most studies have also variably excluded nodules containing coarse macro-calcifications, substantial cystic areas or those within a diffuse thyroiditis as early evidence suggested that USE is suboptimal in these cases. Due to this selection bias, a much higher proportion of nodules in these studies is malignant (~25 %) than in an unselected population (~5 %) and most are papillary carcinomas (~90 %); thus published discriminatory performance data actually refer to this histological type [29–35].

**Fig. 13:**
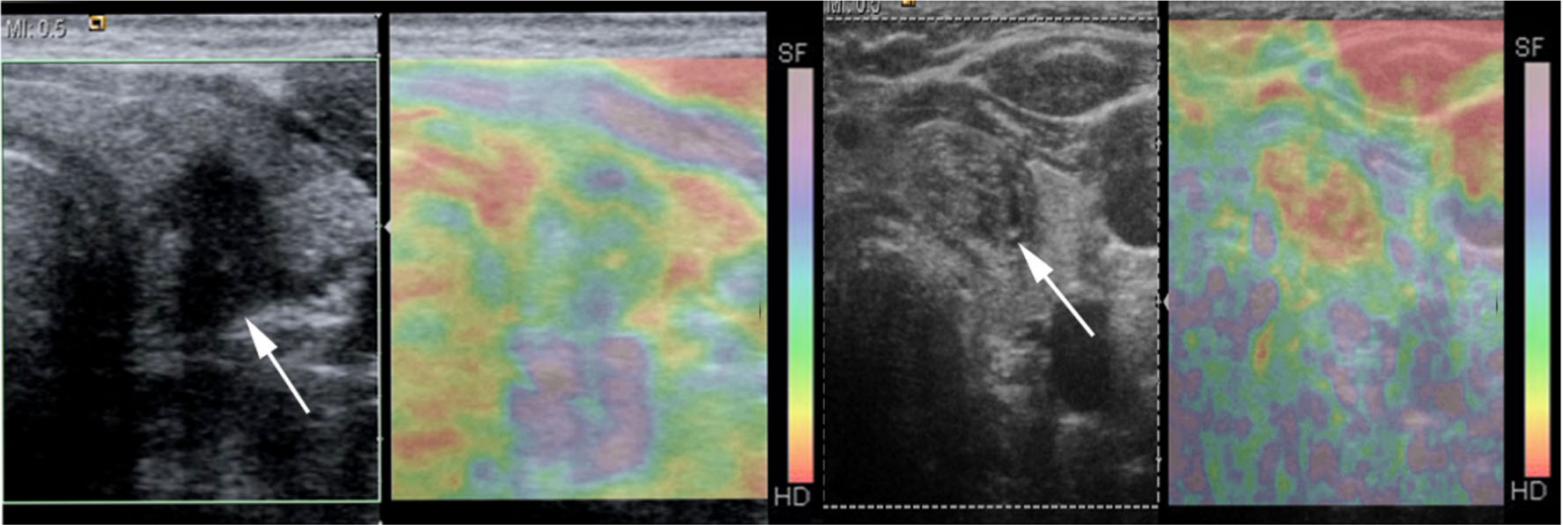
Transverse strain USE with B mode US images of a papillary carcinoma (*left*) and hyperplastic nodule (*right*). Using the qualitative scoring scale from Fig. 11, the cancer appears soft (ES1) whereas the hyperplastic nodule appears mostly stiff (ES3). These would be false negative and false positive for malignancy using most scoring systems published to date, which apply a strain USE threshold of ES3 or greater to predict malignancy

**Fig. 14:**
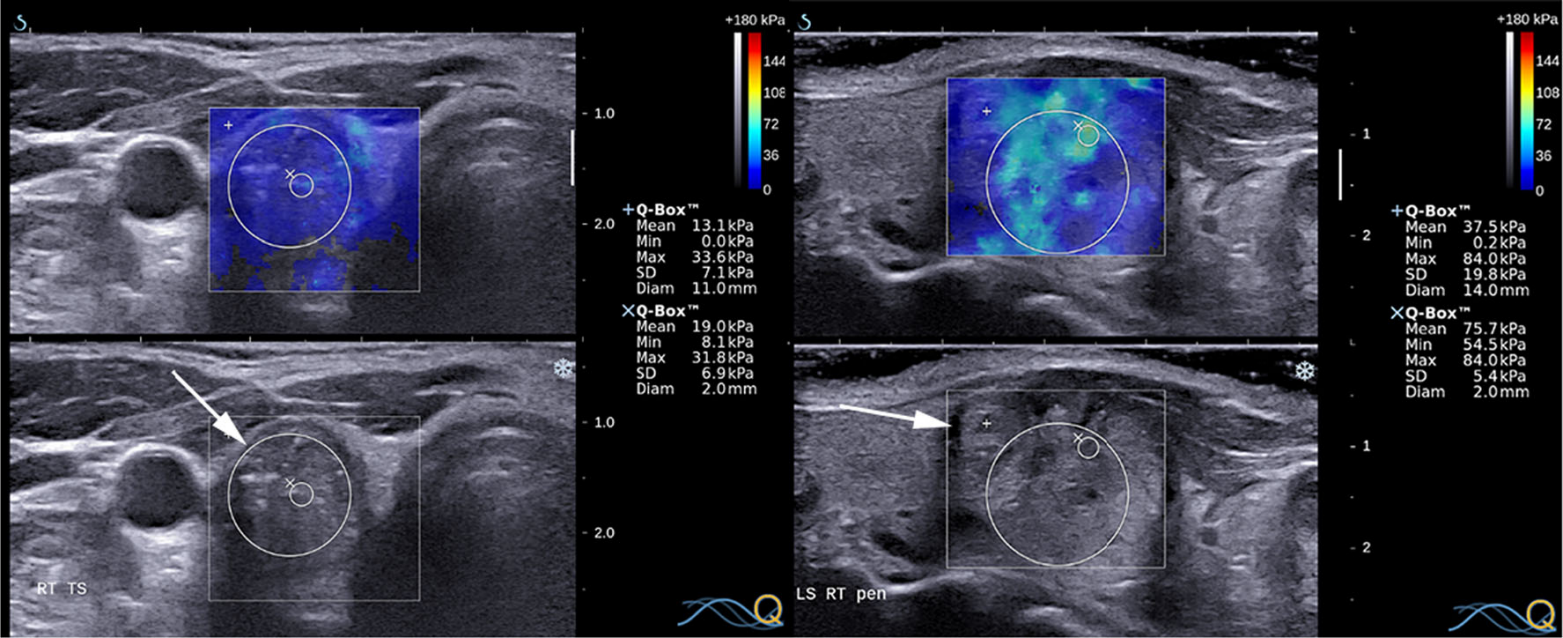
Transverse and longitudinal SWE with B mode US images of a papillary carcinoma (*left*) and a hyperplastic nodule (*right*) respectively. The SWE elastogram chromatic scale ranges from blue to red, denoting soft to stiff (0 kPa to 180 kPa). The cancer appears uniformly soft with a maximum SWE stiffness of 33.6 kPa whereas the hyperplastic nodule appears heterogeneously stiff with a maximum SWE stiffness of 84.0 kPa. These would be false negative and false positive for malignancy respectively using any recently published SWE cutoff (cutoffs range between 39.3–66 kPa)

Promisingly, most studies indicate that papillary cancers have significantly higher stiffness indices on USE compared to benign nodules although there is some overlap (Figs. 11, 12, 13 and 14). Furthermore, most evidence suggests that USE has a superior overall accuracy compared to single or multiple conventional US criteria. Multiple meta-analyses confirm these findings [29–35] including the largest to date, which reports a pooled mean sensitivity, specificity, PPV, NPV and accuracy of 87, 80, 47, 97 and 81 % for strain USE using freehand compression (*N* = 4,926); 87, 80, 52, 100 and 82 % for strain USE using carotid artery pulsation (*N* = 241) and 86, 89, 60, 97 and 89 % for SWE [34]. From the pooled data, SWE has higher accuracy compared to strain USE, while performing strain USE using carotid arterial pulsations is slightly superior diagnostically compared to freehand compression. The precise cause for the high stiffness of papillary cancers needs to be clarified but probably reflects intense tumour fibrosis, with or without calcifications, which is characteristic of this histology although not always present (~80 %) [36]. Indeed, anecdotal examples of high USE indices are reported for benign nodules containing dense fibrotic foci and calcifications histologically as well as low USE indices in some papillary and follicular cancers lacking substantial fibrosis [37].

Despite the mostly promising data described above, a role of USE in thyroid nodules is controversial because of a marked heterogeneity in published accuracy results including optimised cutoffs for malignancy, and importantly a small but significant number of recent studies have markedly suboptimal findings [38–43]. One study assessed 703 nodules by conventional US and qualitative strain USE (31% cancer) and reported a suboptimal diagnostic performance for USE (65% sensitivity, 58% specificity and 61% accuracy) in comparison with conventional US (92% sensitivity, 67% specificity and 74% accuracy) or combined US/USE [42]. Even among studies of strain elastography using semi-quantitative SRs, there is a wide dispersion in mean SRs of benign and malignant nodules as well as optimal cutoffs for malignancy [44–49]. Discrepant results have also been documented for quantitative USE. For example, optimised cutoffs for malignancy in studies using an identical SWE technology range between 39.3 kPa and 66 kPa, and corresponding diagnostic performances range between 52.9 % sensitivity, 77.8 % specificity to 85.2 % sensitivity, 93.9 % specificity [50, 51]. The reasons for the wide variation in results are unclear at present.

- Potential role of use in clinical practice

Approximately a dozen studies have evaluated the combined performance of USE with US (US/USE) [40, 42, 51–59] and nearly one half of these suggest that US/USE can achieve both excellent sensitivity and negative predictive values (NPV) for malignancy (92–100 %), surpassing conventional US [42, 52–56] (Table 1). One study of 498 nodules reported 97 % sensitivity and 97 % NPV for combined US/USE compared to 85 % sensitivity and 91 % NPV for conventional US alone [54]. This is an important finding as it suggests that clinicians may be able to use USE as an ancillary technique to identify nodules with a very low risk of malignancy, which may be placed under observation rather than undergo invasive investigations including FNAC or surgery. Indeed, another study that evaluated 991 nodules using both strain ratios and conventional US within a standardised classification system (US-TIRADS) also documented high sensitivity (98.5 %) and NPV (99.8 %) for combined US-TIRADS/USE and importantly estimated that FNAC could be reduced by 34% using this combined approach [52]. In this respect, the additional value of USE is likely to be greatest for nodules that are indeterminate on conventional US in terms of displaying one or two mildly suspicious features of malignancy, which would all undergo FNAC under the current guidelines. This probably includes nodules classified as TIRADS 3 “probably benign” and possibly TIRADS 4A “low suspicion of malignancy”, which collectively comprise approximately 80% of FNACs and of which 90 % are ultimately benign [60]. USE is unlikely to be useful in terms of altering the clinical decision of whether to perform FNAC for nodules that are already very suspicious for malignancy or completely benign on conventional US.

**Table 1:** Diagnostic performances of conventional US and a combination of conventional US and USE for diagnosing thyroid malignancy

First author, year (citation)	n (% malignant)	Type	Sensitivity (%)		Specificity (%)		Accuracy (%)		PPV (%)		NPV (%)	
			US	US/USE	US	US/USE	US	US/USE	US	US/USE	US	US/USE
Sebag, 2010 [46]	146 (19.9)	SWE	51.9	81.5	97	97	ND	ND	82.4	88	88.1	95
Trimboli, 2012 [49]	498 (25.3)	SE	85	**97**	54	34	62	50	38	33	91	**97**
Ragazzoni, 2012 [52]	132 (30.3)	SE	70	85	92.4	83.7	85.6	84.1	80	69.4	87.6	92.8
Cappelli, 2012 [51]	159 (9.4)	SE	80	**100**	75	70.8	75.4	73.6	25	26.3	97.2	**100**
Moon, 2012 [37]	703 (30.8)	SE	91.7	92.2	66.7	65	74.4	73.4	55.1	54.1	94.7	94.9
Unluturk, 2012 [35]	237 (24.5)	SE	69	41	85	93	81	81	60	67	89	83
Veyrieres, 2012 [50]	297 (11.8)	SWE	77.1	**97.1**	58	55.3	ND	ND	19.7	22.5	95	**99.3**
Shweel, 2013 [48]	66 (24.2)	SE	92	**95.4**	72.9	94.8	60.1	95.2	95	82.3	63.1	**98.8**
Russ, 2013 [47]	991 (6.7)	SE	95.7	**98.5**	61	44.7	62	48.3	ND	ND	99.7	**99.8**
Garino, 2014 [54]	108 (30.6)	SE	61	88	83	77	76	81	61	63	83	94
Liu, 2014 [53]	331 (30.5)	SWE	76.2	87.1	83	73.9	81	78	66.4	59.5	88.8	92.9

Only published studies with both documented sensitivity and NPV results are shown, and those with high sensitivities and NPVs (>95%) are indicated in bold. *US* conventional US, *PPV* positive predictive value, *NPV* negative predictive value, *US/USE* combined conventional US and USE, *SE* strain USE, *SWE* shear wave elastography, *ND* not documented

Another potential role for USE is for nodules with non-diagnostic or indeterminate cytological results on FNAC (e.g., cellular atypia of indeterminate significance), which affects approximately 30% of FNACs [61–63]. A substantial proportion of these nodules undergo hemi-thyroidectomy for a definitive diagnosis because repeat FNACs are non-diagnostic or because of limitations in cytological diagnosis of follicular lesions, although 75–80% of these are ultimately non-neoplastic [61, 62, 64]. Encouragingly several reports indicate that USE or combined US/USE can attain high NPVs for malignancy in this group (95–100 % NPV) [56, 59, 65, 66], although one study has documented the opposite findings (50% NPV) [39]. If these positive findings can be validated in larger studies, USE could lower the number of hemi-thyroidectomies performed in this subgroup.

- Potential limitations of USE

Unfortunately, follicular carcinomas appear to have low mean USE indices that overlap substantially with benign hyperplastic nodules and follicular adenomas [33]. This is disappointing given the challenges in pre-surgical diagnosis of follicular lesions but unsurprising given their histological similarities [67]. Sparse data also suggest that USE is suboptimal for detecting medullary thyroid carcinomas [68]. Macro-calcifications are a confounding factor for USE as they increase mean stiffness estimates of ROIs that include them and are common in both benign and malignant pathologies [69, 70]. However, some evidence suggests that novel semi-quantitative USE indices such as strain heterogeneity may be diagnostically accurate in partially calcified nodules [70]. Predominantly cystic nodules are unsuitable for strain USE but not SWE for technical reasons related to uneven stress transmission in the former [28, 41]. Graves disease, Hashimoto’s thyroiditis and subacute thyroiditis can cause parenchymal stiffening [71–73] and therefore would be expected to lower the accuracy of strain USE for malignancy, although some evidence suggests that strain USE is still accurate for nodules in a background thyroiditis [66, 74]. As SWE does not require a reference tissue, nodule stiffness estimated by SWE is probably not altered significantly by a background thyroiditis [69, 75]. The influence of nodule dimensions on USE indices is controversial although USE appears to have similar diagnostic performances for nodules ranging between 1 and 3 cm in diameter. Some evidence indicates that USE indices and diagnostic accuracies are reduced in infra-centimetric nodules, although many reports have found USE still to be highly accurate in this subgroup [58, 70, 76, 77].

- Practical aspects and operator dependence of USE

USE is a dynamic technique with fluctuating elastographic appearances due to its intrinsic sensitivity to miniscule displacements that are continually occurring under physiological conditions. Various components of USE acquisition and interpretation are operator dependent including freehand compression for strain USE, selection of imaging planes through nodules, selection of representative elastograms, qualitative scoring of elastograms and placement of ROIs for (semi-)quantitative measurements [78]. Strain USE using freehand compression is highly dependent on an optimal axial compression technique, which is hampered by competing non-axial motions (e.g., arterial pulsations and respiration) and the different mobilities of tissues bordering the thyroid (e.g., immobile trachea and mobile vessels). Carotid artery strain USE and SWE circumvent this source of operator dependence. Nevertheless, irrespective of the USE technology used, the operator influences the amount of resting pressure applied via a motionless transducer, termed precompression, which in turn can alter tissue stiffness [34]. This phenomenon reflects the fact the Young’s modulus of biological tissues increases as strain is increased, termed strain hardening, although this effect is minimal for very small strains (<10 %). To mitigate this problem, USE should be performed with minimal pressure applied, which may be facilitated by maintaining an ample US gel layer between the transducer and skin.

**Fig. 15:**
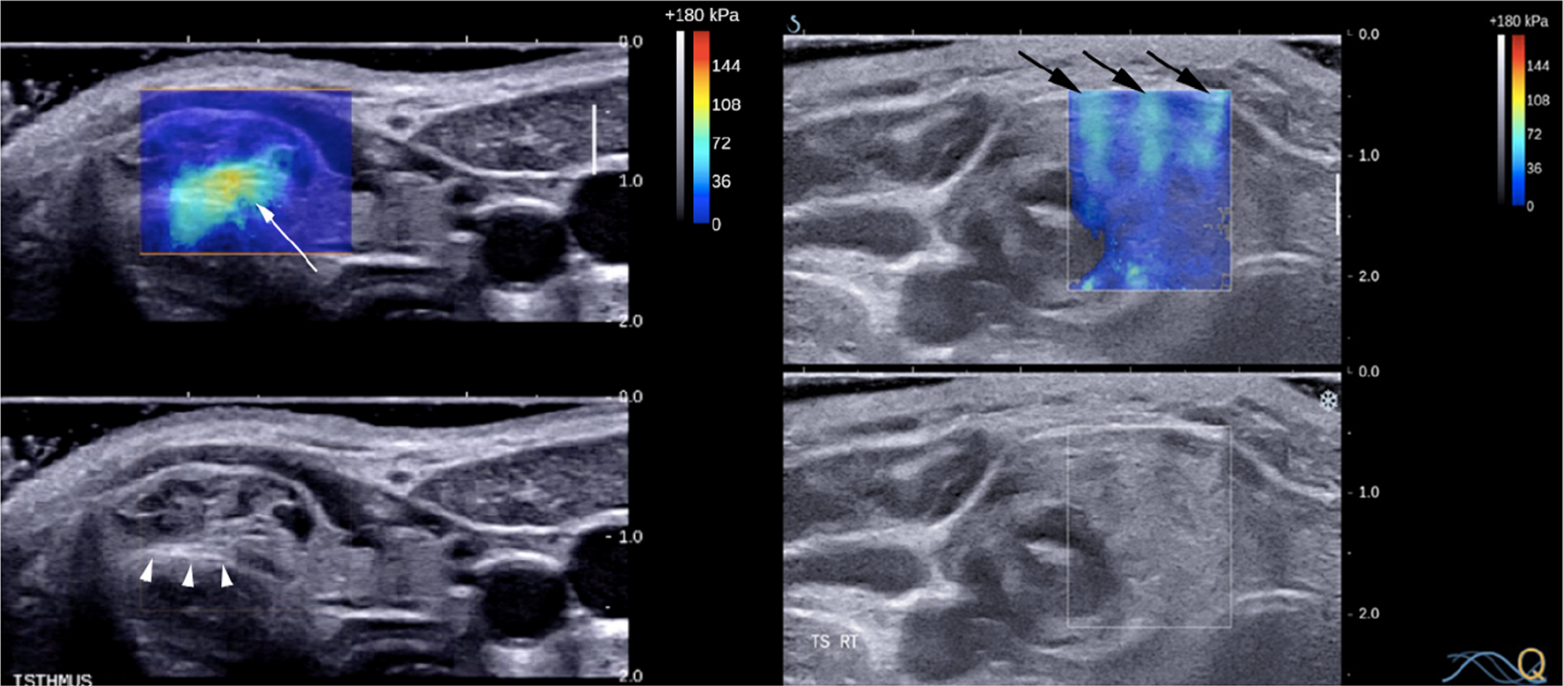
Transverse SWE with B mode US images of two hyperplastic nodules (*left, right*) showing elastographic artefacts. The SWE elastogram chromatic scale ranges from blue to red, denoting soft to stiff (0 kPa to 180 kPa). The left image highlights stress concentration artefact in an isthmic nodule (*white arrow*), which occurs because the nodule produces a convex bulge of the skin and is compressed against the relatively hard trachea despite the use of an ample gel layer on the skin surface. The right image shows vertically aligned bands of light blue colour that traverse several anatomic structures (*black arrows*). These are suggestive of technology-related push pulse artefacts corresponding to peaks of shear waves induced at different lengths along the transducer. Although they can be excluded from SWE measurements, they may be inadvertently included if subtle

Various elastographic pitfalls and artefacts can occur in thyroid USE, which need to be recognised and avoided. High stiffness areas due to the stress concentration frequently develop in the near field beneath the transducer including the superficial aspect of some thyroid nodules, as well as at boundaries of tissues with contrasting stiffness including the margins of some nodules. Isthmic nodules are especially prone to stress concentration effects because they tend to produce a focal skin bulge with uneven contact under the flat transducer and be compressed directly against the unyielding trachea. Other artefacts can also develop because of a suboptimal compression technique, competing non-axial motions, presence of cystic fluid and limitations of specific USE technologies (Fig. 15) [25, 79].

- Reproducibility of USE

**Fig. 16:**
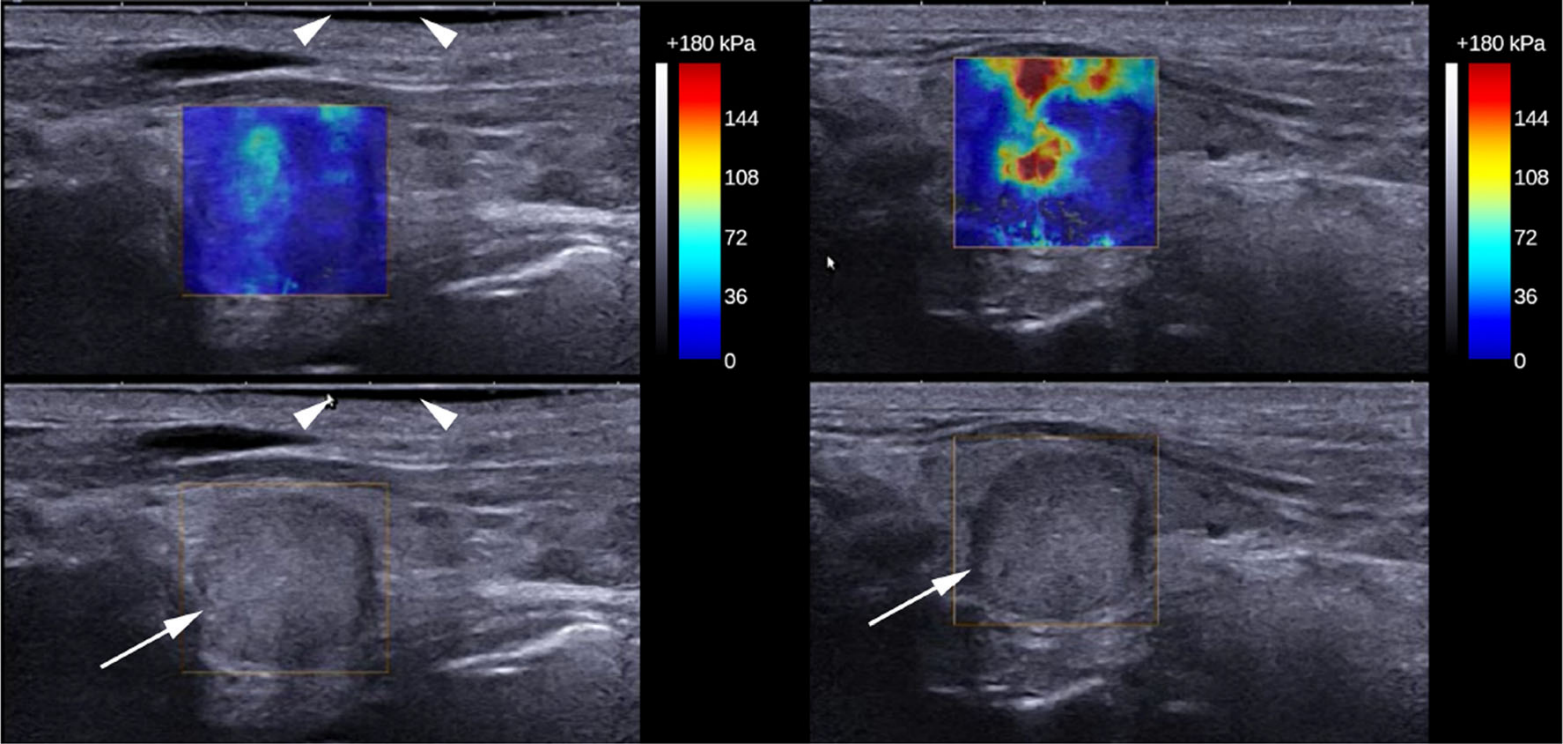
Longitudinal SWE with a B mode US image of a papillary carcinoma under different degrees of precompression applied by the operator. The SWE elastogram chromatic scale ranges from blue to red, denoting soft to stiff (0 kPa to 180 kPa). The left image was acquired using minimal precompression as evidenced by a preserved gel layer between the skin and transducer (*arrowheads*), whereas the right image was acquired using mild precompression as evidenced by the fact that the gel interface layer is effaced and the nodule is closer to the skin surface. The SWE stiffness of the cancer is much higher in the right image. This deliberate example illustrates how variations in precompression can bias USE results

Given the aforementioned sources of operator influence, practical challenges and artefacts, establishing a high reproducibility of thyroid USE is an important objective. An early report of strain USE had disappointing reproducibility results [80]; however most recent studies using newer USE technologies report substantial to excellent intra- and inter-observer agreements [49, 55, 57, 79, 81–85]. Evidently, the inclusion of compression quality assurance scales on strain USE systems has had a positive influence on this aspect. Nevertheless, the fact remains that (semi-)quantitative USE indices have differed substantially between studies, including those evaluating identical SWE technologies [35]. These discrepancies may reflect sampling variations although their magnitude and pattern raises questions about additional mechanisms, which in turn may influence reproducibility. One postulation is that operators may be performing USE using different degrees of precompression (Fig. 16) and may even be unconsciously influenced by the nodule's grey-scale appearances on the split-screen display (cognitive bias) [86, 87]. At present, precompression cannot be measured or standardised reliably because pressure sensors are not incorporated within transducers.

- Current and future directions of USE

According to recently published guidelines from the European Federation of Societies of Ultrasound in Medicine and Biology (EFSUMB): “elastography is an additional tool for thyroid lesion differentiation” and “based on expert opinion, elastography may be used to guide follow-up of lesions negative for malignancy at FNAC” [88]. This cautious endorsement reflects the current controversies in thyroid USE evidence. Clearly further research is required including large multi-centre prospective trials of nodules with less pre-selection to evaluate different nodule characteristics, uncommon pathologies and reproducibility and ultimately determine how USE can be integrated with conventional US into emerging malignant risk classification systems (e.g., TIRADS). In this respect, clarifying precisely which types of nodule are unsuitable for thyroid USE will be critical to optimising its accuracy.

Although controversial, USE shows promise as an adjunct to conventional US for reliable identification of benignity in a select group of nodules on the initial US examination as well as those with non-diagnostic or indeterminate cytological results following FNAC. Even if these indications are validated in future studies, several issues will still need to be addressed before USE can be accepted widely in routine practice. USE technologies should be robust and simple to apply by the range of health care professionals who perform thyroid US routinely. The specific strengths and limitations of each USE technology including artefacts need to be catalogued systematically. While it is acknowledged that USE technologies are proprietary and their outputs may not be interchangeable, there should be greater standardisation of thyroid USE, for example by unifying nodule-grading systems and elastogram chromatic scales. Fortunately, USE technologies are continually improving including with respect to measurement precision and quality assurance, which should augment their diagnostic accuracies and reliabilities. Ideally, with advances in CAD software, a nodule’s grey scale and elastographic features could be analysed automatically and converted into a single estimate of its malignant risk. There are still some hurdles to overcome although, at the current pace of promising research and technological advances, USE may well become a valuable complementary technique for evaluating thyroid nodules in the near future.

## Conclusion

Innovative advances in high-resolution ultrasound now enable detailed anatomical characterisation and accurate differentiation of benign from malignant disease. Ultrasound has become the core component of thyroid nodule guidelines, but it is important that the key technological aspects of the modality are understood and that challenges and limitations remain with the technique. The large evidence base for ultrasound elastography indicates that the assessment of nodule stiffness can improve the imaging evaluation of thyroid lesions and potentially avoids unnecessary FNAC/surgery for benign nodules, particularly if integrated with US classification. The potential for 3D and CEUS in thyroid nodule US has not yet been realised, but the emergence of these novel approaches is an illustration of the huge strides that have been made in the underlying ultrasound technology.
